# Reconciling the science and policy divide: The reality of scaling up antiretroviral therapy in South Africa

**DOI:** 10.4102/sajhivmed.v16i1.355

**Published:** 2015-07-02

**Authors:** Alan Whiteside, Jamie Cohen, Michael Strauss

**Affiliations:** 1 Balsillie School of International Affairs, Waterloo, Canada; 2 College of Law and Management Studies, University of KwaZulu-Natal, South Africa; 3 Harvard School of Public Health, Boston, United States; 4 Health Economics and HIV and AIDS Research Division, University of KwaZulu-Natal, South Africa

## Abstract

With the world’s largest national treatment programme and over 340 000 incident cases annually, the response to HIV in South Africa is hotly contested and there is sometimes a dissonance between activism, science and policy. Too often, policy, whilst well intentioned, is informed only by epidemiological data. The state of the healthcare system and sociocultural factors drive and shape the epidemic and its response. By analysis of the financial, infrastructural, human resources for health, and governance landscape in South Africa, we assess the feasibility and associated costs of implementing a universal test and treat programme. We situate a universal test and treat strategy within the governance, fiscal, human resources for health, and infrastructural landscape in South Africa. We argue that the response to the epidemic must be forward thinking, progressive and make the most of the benefits from treatment as prevention. However, the logistics of implementing a universal test and treat strategy mean that this option is problematic in the short term. We recommend a health systems strengthening HIV treatment and prevention approach that includes scaling up treatment (for treatment and prevention) along with a range of other prevention strategies.

## Introduction

South Africa currently has the world’s highest national incidence of HIV and AIDS, with 6.4 million people infected and over 340 000 incident cases annually.^1^ The history of the epidemic and the political, economic and policy response has been well documented by academics.^2,3,4^ In brief, the government refused from 1999 to 2004 to provide antiretroviral therapy via public facilities. The then Head of State, Thabo Mbeki, aided and abetted by Minister of Health Manto Tshabalala-Msimang, a few members of his cabinet and some of the provincial governments, questioned the causal link between the HI virus and AIDS, the effects of antiretroviral therapy (ART), and the motives of pharmaceutical companies.^5^ Despite this opposition, drugs were made available by progressive provincial governments, health workers and activist organisations. Since those bleak days of AIDS denialism, and increasingly from 2005,^5^ South Africa has expanded its HIV services and today has the world’s largest ART programme, reaching about 2.1 million people.^1^

Like most countries in sub-Saharan Africa, South Africa tries to base its health policy on World Health Organization (WHO) recommendations. Their guidelines are based on the best available scientific evidence, but do not always speak to economic and social realities, especially regarding HIV and AIDS. The first WHO HIV treatment guidelines were released in 2002, and updated in 2006, 2010 and 2013. The latest WHO clinical recommendations promote treatment initiation for adults and adolescents > 10 years old whose CD4 count falls below 500 cells/mm^3^ and immediate treatment for persons with active TB disease; hepatitis B virus (HBV) co-infection with severe chronic liver disease; pregnant and breastfeeding women with HIV; and those who are HIV-positive in a serodiscordant partnership.^6^

The South African National Department of Health adopted the 2010 recommendations to start adults on treatment at a CD4 cell count ≤ 350 cells/mm^3^, and in 2015 partially adopted the new guidelines.^7^ The only WHO recommendation not adopted in the new South African ART guidelines is the recommendation to initiate serodiscordant couples regardless of CD4 count; the discussion of whether or not to include this is ongoing. However, the government is hesitant to start all HIV-infected individuals on treatment irrespective of CD4 level.

Historically, WHO recommendations drive policy. In the light of the delayed and discrepant policy change in South Africa, it is worth examining the WHO recommendations and process with careful consideration of context. Whilst scientific evidence provides the foundation for the WHO’s guidance on expanded treatment eligibility, scientific literature tends to be produced in a vacuum. Increasing the CD4 threshold has implications that reverberate across sectors: it affects budgets, infrastructure and human resources.

In the present article, we examine how policy has changed and existing constraints on the South African government. The policy debate has been articulated in the pages of this journal, but it is noteworthy that this is the only place where we have seen these arguments^7,8^ as they are generally seen as politically incorrect and going against international norms.

We argue that policymakers must consider the possibility that a ’one-size-fits-all’ approach to treatment may not be the most beneficial for patients’ health and adherence to treatment, or the most cost-effective for the national budget.^7^ Decisions to change the CD4 threshold in South Africa are not taken lightly, but we are not convinced that there has been sufficient consideration of the implications regarding capacity, adherence and the health of the population. We argue that the treatment agenda has been set outside the country and to unrealistic levels reflecting a dissonance between activism, science and policy.^9^

The success in translating evidence into practice depends on local context, resources, priorities and data. It is our view that the South African government’s position is that it, reluctantly, has to change policy if not practice. This works for a relatively well-off country with a powerful political leadership, reasonable public health system, low (and falling) dependence on donors, and a reasonable public platform for debate. In many other settings, governments and ministries of health may be adversely affected by the decrees from Geneva or global capitals.

The present article identifies two major challenges faced in South Africa. First, modelling studies are being given too much emphasis and should not be used, in isolation, to dictate policy. Second, the issue of when to provide ART is being conflated with treatment-based prevention.

It is important, however, to understand the two pressures for treatment. First, it is seen by some as a ‘right’ that HIV-positive people should get the best available treatment, without considering the broader health context and the need to make choices. Second is the pressure of treatment as prevention. The seminal HPTN 052 study showed the effectiveness of ART in reducing HIV transmission in discordant heterosexual couples.^10^ This study was a key driver for the WHO to revise its guidelines for ART initiation.^11^ ART is recommended for HIV–positive, pregnant, breastfeeding women; all children under the age of five; people with TB or hepatitis B; and HIV–positive people with HIV-negative partners.^6^

For clarity, we include working definitions of Universal Access and Universal Test and Treat ART rollout strategies as well as treatment as prevention (TasP) (see panel). Papers^12,13,14,15^ that model the cost-effectiveness of universal test and treat in South Africa, using various inputs and assumptions, yield different results. We include an overview of the findings and conclusions of these studies to show the different predicted outcomes and costs of universal test and treat.

Models are a mixed blessing – they can generate useful cost-effectiveness data if they are based on realistic assumptions and up to date biomedical data. However, achieving this often proves to be difficult, especially for long-term analyses, because even small inaccuracies or unforeseen costs may lead to very different results. Meyer-Rath and Over^15^ warn about the shortcomings of ART scale-up models that do not account for the flexibility of costs over time. Their model showed that small-scale inefficiencies could lead to far higher costs in the long run.

Although prevention necessarily results from any treatment programme,^16^ it is important when developing policy not to conflate TasP with universal test and treat. We critique universal test and treat in the context of governance, human resources for health, and infrastructural and fiscal constraints in South Africa. The response to the epidemic must be forward thinking, progressive and to make the most of the benefits from TasP. The logistics of implementing a universal test and treat strategy mean that this option is problematic in the short term. We recommend a health systems strengthening HIV treatment and prevention approach, which includes scaling up treatment (for treatment and prevention) along with a range of other prevention strategies.

## Governance

South Africa’s Constitution established three spheres of government–national, provincial, and local. Health policy is developed nationally and adapted to fit provincial needs. Healthcare funding, including that for HIV and AIDS, is allocated to provincial departments of health. This system has created and exacerbated health inequities between provinces. The political, socioeconomic and historical context in the nine provinces varies significantly, which affects service delivery (see Table 1).^17^ We illustrate this point with data from the best and worst provinces: the well-resourced and well-governed Western Cape, and Limpopo which has a largely rural population, fewer human resources per capita and inefficient government.

**TABLE 1:** Provincial inequality.

Indicator	Limpopo	Western Cape
Contribution to gross domestic product (2010)	7.2%	14.1%
Percentage of total population	10.4%	11.2%
Percentage of rural population	90%	10%
HIV prevalence (ages 15–49)	12.92%	4.75%
Number of people living with HIV	409 161	273 114
Number of private hospitals	8	34
Number of public hospitals	42	55
Number of public sector doctors per 100 000 population	20.7	34.8
Health professional vacancy rate	58.5%	29.0%

Data compiled from the public domain.

The health profile in South Africa’s nine provinces varies between these two extremes. HIV prevalence ranges from 4.75% in the Western Cape to 24.7% in KwaZulu-Natal – nearly double that in Limpopo. The number of annual new HIV infections varies by a factor of 30.^18^ In this context, one-size-fits-all interventions cannot work; the gaps in health service facilities, especially staffing levels, are too great. A blanket universal test and treat strategy would be difficult to apply.

## Human resources for health capacity

Human resources are a significant limitation on the ability to effectively deliver health and HIV services. Progress has been made; the number of public sector doctors increased from 7645 in 2003 to 13 614 in 2013, and the number of professional nurses registered with the South African Nursing Council from 96 715 to 129 015.^19^ The number of doctors and professional nurses per 100 000 people exceeds the WHO minimum.

**BOX 1:** Definitions and models.

Definitions
**Universal access:** Treating 80% of HIV-infected individuals with CD4 count < 350 cells/μL, offering ART for discordant couples regardless of CD4 count^32^
**Universal test and treat:** Treating all HIV-infected individuals irrespective of CD4 count^13^
**Treatment as prevention:** HIV prevention methods that use ART in HIV-positive persons to decrease the chance of HIV transmission independent of CD4 count^11^
**Models estimating the cost and cost-effectiveness of universal test and treat in South Africa**
Granich R, Kahn JG, Bennett R, et al. Expanding ART for treatment and prevention of HIV in South Africa: Estimated cost and cost-effectiveness 2011–2050. PLoS ONE 2012; 7:e30216.
Findings: Model predicts universal access (at CD4 < 350 cells/μL) would reduce new HIV infections by an estimated 265 000 over 5 years and 1.4 million over 40 years. Universal access could reduce estimated deaths by 200 000 and save US$504 million over 5 years. Over 40 years, it could reduce deaths by 2.9 million, DALYs by 15.7 million, and costs by $3.9 billion. Achieving universal test and treat would result in a further decline of 3.3 million infections, 3.5 million deaths, 25.7 million DALYs, and would cost $10 million less over 40 years, compared with achieving universal access. Conclusion: A universal test and treat programme is an optimal implementation of TasP.
Wagner BG, Blower S. Universal access to HIV treatment versus universal ‘test and treat’: Transmission, drug resistance and treatment costs. PLoS ONE 2012;7:e41212.
Findings: Model finds that a universal test and treat strategy could eliminate HIV after 40 years and would cost $12 billion more than achieving universal access. Achieving universal access would prevent 4 million infections after 20 years and 11 million after 40 years. Conclusion: A universal access programme is a better implementation of TasP than universal test and treat.
Meyer-Rath G, Over M. HIV treatment as prevention: Modelling the cost of antiretroviral treatment—state of the art and future directions. PLoS Med. 2012;9: e1001247. http://dx.doi.org/10.1371/journal.pmed.1001247
Findings: Many existing models of TasP and ART scale-up do not use realistic assumptions about costs and cost structures, and therefore cannot accurately predict costs – especially in the long term. Conclusion: A universal test and treat programme in South Africa will cost 42% more than the cost predicted by the WHO model.
Bärnighausen T, Bloom DE, Humai S. Economics of antiretroviral treatment vs. circumcision for HIV prevention. PNAS. 2012;109:21271–21276.
Findings: Model finds universal access combined with high medical male circumcision coverage provides approximately the same HIV incidence reduction as universal test and treat, for $5 billion less over 2009 – 2020. Conclusion: Universal access and high MMC coverage is a better combination prevention strategy than universal test and treat, and better use of the benefits of TasP.

The problem is the uneven distribution of human resources for health (HRH) across provinces; between urban and rural areas; and between the public and private sectors.^19^ Only 12% of the country’s doctors and 19% of its nurses work in rural facilities, serving 43.6% of the population.^20^ These additional strains on an already overstretched health labour force compromise the ability of the health system to provide equal access and increased coverage of treatment and care for people with HIV.

Health facilities face other human resource problems. The real unit costs of labour have almost doubled over the last 15 years.^19^ Put simply, this means that compensation has gone up more than productivity, hence more money has to be spent to achieve the same result. Attrition owing to AIDS and emigration has worsened the ratio of professional and enrolled nurses and doctors to population size in the public sector. There is an historical trend of a substantial proportion of doctors who qualified in South Africa emigrating to work abroad, and many leave the public sector to work in private practice.^20^

Responses include expanding and auditing the quality and relevance of training to meet the demand for health services, task shifting and developing strategies for ensuring an equitably distributed, long-term supply of HRH. Government’s training targets may enable South Africa to reach universal ART, if the skewed distribution of HRH is addressed in conjunction with innovative, task shifting measures. Building human resource capacity to meet the needs of a universal test and treat programme will be a significant challenge in South Africa and will need to be an increasing priority as the treatment programme expands.

## Infrastructural capacity

South Africa’s healthcare infrastructure has improved since 1994, but regional and sectoral discrepancies persist (Table 1). Fifteen per cent of poor rural households live more than one hour away from the closest clinic, and 20% live more than one hour away from the closest hospital.^21^ The fragility of the health system was clearly illustrated in December 2012 when Médecins Sans Frontières had to step in to supply the Mthatha medical depot with ARVs for 50 000 HIV-infected patients. An estimated 5494 adults taking ARVs went at least one day without treatment owing to severe drug supply and delivery disruptions.^22,23^ This service delivery crisis was a symptom of long-standing and systemic infrastructural, human resource capacity and financial mismanagement issues, which continue today.^24^

A universal test and treat programme would place strain on a fragile system, possibly particularly compromising rural healthcare where there may be fewer staff members, and also a dependence on urban-based supply chains. South Africa’s public health infrastructure must be strengthened to support the demands of universal ART and the proposed National Health Insurance (NHI) plan.

## Financial capacity

A good understanding of South Africa’s healthcare spending shows additional constraints. Overall expenditure on public health grew from R16.4 billion in 2008/2009 to over R30 billion in 2013/2014.^25^ A key component of the expanded health budget is the NHI plan, to be phased-in over 14 years. This plan aims to restructure and strengthen the public health system and improve quality of care. The NHI will require significant financial expenditure, imposing a further fiscal burden on taxpayers. NHI-related public spending increased from R119.3 million in 2008/2009 to R491.8 million in 2013/2014, and is expected to increase to R650.1 million by 2016/2017.^25^

This expense must be seen against a backdrop of rising HIV spending. Public spending on HIV and AIDS increased from R3.36 billion in 2008/2009 to R10.97 billion in 2013/2014.^25^ Clinical care and treatment support from the President’s Emergency Plan for AIDS Relief (PEPFAR), South Africa’s largest bilateral donor, will come to an end in 2017, and the focus of this funding is already shifting from treatment to support and technical assistance. The government committed to increase its financing of the National Strategic Plan for HIV, STIs and TB (NSP) from R9.57 billion in 2012 (71% of NSP) to R15.2 billion in 2017 (88% of NSP).^26^ Despite this outlay, the government is forecasting a widening funding gap; in 2013/2014 it was R2.67 billion. By 2015/2016 the projections are: resource needs of R29.86 billion, government funding of R19.8 billion, and development partners providing R5.02 billion – leaving a gap of over R5 billion.^27^

A universal test and treat strategy does not currently fit into South Africa’s available financial resources and spending priorities. Although NHI expenditure will strengthen HIV services, it crowds out the feasibility of investment in a universal test and treat programme unless significant new money is allocated; this could come from re-allocation of national budgets but would require political will.

## Treating the region?

There is evidence of an increasing burden on existing health resources because of ’medical tourists’ in South Africa, especially from neighbouring countries. In 2012 there were 6 689 105 African or Middle Eastern visitors to South Africa, of whom 260 875 or 3.9% were defined as medical tourists. Not all are seeking public health; middle-class visitors may be coming for elective procedures or be covered by insurance; and some regional governments refer (and pay for) patients to attend specialised medical facilities and this is covered under the 1999 Southern African Development Community (SADC) Health Protocol.^28^

There is evidence to show that, as ART coverage improves across the border, so the number of people trying to access treatment in South Africa declines. In the case of Botswana, medical tourists grew from 40 000 to just over 50 000 between 2003 and 2008. Thereafter, the numbers fell to less than 20 000 in 2012. For Mozambique, the number of medical tourists grew rapidly from 8000 in 2003 to about 180 000 in 2010. With the expansion of ART coverage in Mozambique, the numbers fell and in 2012 were just less than 100 000.^28^ The exception may be Zimbabweans who are primarily in South Africa for employment, and who are accessing ART. Of course, there is also evidence that South Africans are making it difficult for visitors to access treatment in the public sector, and the appalling April 2015 xenophobic attacks sent a message that foreigners are not welcome in the country for any reason. Nevertheless, providing ART to all makes public health sense.

## The HIV endgame: An AIDS-free generation

Significant scientific advances coupled with political will have endowed the world with effective biomedical, behavioural and structural interventions. The conversation is shifting towards a foreseeable end to the epidemic. In the absence of a vaccine or cure, navigating the ‘endgame’ will be costly and ineffective if plans are designed without context and foresight.

The HIV epidemic in South Africa is driven by deeply entrenched social and structural factors including gender inequality, poverty and migration.^29,30^ Even if the structural inadequacies of the healthcare system are addressed, a one-size-fits-all approach to HIV treatment and prevention will not work. The complexity of the epidemic necessitates a diverse range of interventions which are effective within the South African epidemiological and resource context.

Too often, health policy recommendations are prescribed with good intention but based primarily on modelled outcomes or international ‘norms’, with little attention paid to local context. Successful programmes that will make a real impact hinge on much more. Fiscal, infrastructural and human resources for health, political and sociocultural factors drive and shape the epidemic and should inform its response.

Given these constraints, it is clear that a universal test and treat strategy, and a combination prevention and treatment approach that includes a strengthening of the health system, are mutually exclusive in the short term. A premature move to a one-size-fits-all policy will undermine the importance of other interventions that work. Rolling out a comprehensive treatment and prevention programme for HIV based on evidence for efficacy, cost-effectiveness and country-specific context must become a priority.

Strengthening the health system and increasing capacity for HIV treatment must remain high on the agenda, but a universal test and treat strategy cannot be pursued exclusively or to the detriment of other interventions that work, as we have shown elsewhere that there is the potential for a virtuous cycle to begin but it must be properly planned.^31^

In addition to medical interventions, there must be increased emphasis on gender relations and empowering women, especially young women. There needs to be social change, and it may be that requiring some form of payback for treatment – not financial but perhaps in terms of volunteerism – will contribute to this.

## Conclusion

South Africa is achieving significant progress in the form of dramatically lower rates of mother-to-child-transmission, falling new infections in young adults, decreasing HIV-related mortality, and expanding ART coverage. We cannot risk derailing this momentum with premature policy decisions. Universal access should be the primary goal and, in this context, treatment will necessarily lead to prevention.
